# Nutrition at the Frontier of Allergy—From Oral Challenges to the Microbiome

**DOI:** 10.3390/nu18081207

**Published:** 2026-04-11

**Authors:** Adam J. Sybilski, Weronika Balas

**Affiliations:** 1 Clinical Department of Paediatrics and Allergology, National Medical Institute of the Ministry of the Interior and Administration, 02-507 Warsaw, Poland; stefa.skup@gmail.com; 2 Second Department of Paediatrics, Centre of Postgraduate Medical Education, 01-813 Warsaw, Poland

Food allergy and related allergic conditions represent a growing public health challenge. Prevalence estimates suggest that IgE-mediated food allergy affects approximately 8% of children and nearly 10% of adults in high-income countries, yet the gap between self-reported and objectively confirmed disease remains substantial [1]. Against this backdrop, the relationship between nutritional exposures, dietary patterns, and the allergic immune response has emerged as one of the most dynamic and clinically consequential areas in allergy research [2]. This Special Issue, Exploring the Role of Nutrition in Modulating Allergies and Allergic Reactions, collates five original contributions that collectively advance our understanding of food allergy diagnosis, risk stratification, disease management, and the emerging biological interface between the gut microbiome and immune tolerance.

The five publications span a broad yet coherent intellectual arc: from the bedside realities of oral food challenge (OFC) performance and its predictors of failure, through to the clinical complexity of eosinophilic esophagitis (EoE) overlapping with IgE sensitization, to the niche but clinically important phenomenon of non-cow mammalian milk allergy, and finally to two papers addressing systemic modulators of allergic reaction severity—nutritional status in pediatric anaphylaxis and the gut microbiome as a target for food allergy therapeutics. Together, they highlight both what is now understood and where the field must go.

## 1. Gaps in Knowledge That This Special Issue Addresses

A foundational gap addressed by this collection is the need for better tools to predict who will fail an OFC and who can safely undergo one. Despite OFCs remaining the gold standard for food allergy diagnosis, their performance in heterogeneous clinical populations is inconsistently characterized. Klim et al. contribute a single-center retrospective analysis of 205 OFCs in Polish children, finding an overall failure rate of 32.2% and identifying asthma and multi-food allergy as independent risk factors for OFC failure. Their ROC-derived specific IgE (sIgE) cut-offs—58.1 kU/L for baked cow’s milk challenges (AUC 0.77) and 11.3 kU/L for baked hen’s egg (AUC 0.66)—represent population-specific thresholds that complement, but do not replace, clinical judgment. Crucially, they confirm the safety of OFCs in a supervised hospital setting, with severe multisystemic reactions occurring in fewer than 2% of procedures. This study directly addresses the practical clinical need for evidence-based patient selection criteria prior to food challenges, filling a gap in the European pediatric OFC literature.

A second underexplored question is whether body weight modulates the severity of allergic reactions in children as it demonstrably does in adults. Obesity is a recognized risk factor for more severe and even fatal anaphylaxis in adult populations, yet pediatric data have been sparse. Kucharek et al. address this directly through a retrospective analysis of 199 hospitalized children stratified by BMI category. Their finding—that nutritional status did not predict anaphylaxis severity or organ system involvement—is itself clinically significant, cautioning against uncritically extrapolating adult risk frameworks to children and raising the hypothesis that obesity’s amplifying effect on anaphylaxis severity may be age-dependent, becoming clinically relevant only after years of metabolic and cardiovascular comorbidity accumulate. The absence of a signal here is as informative as a positive finding would have been.

A third gap lies at the intersection of EoE and IgE-mediated food allergy—a dual diagnosis that is increasingly recognized in children but for which prospective, real-world data on quality of life (QoL) impact remain scarce. Nuyttens et al. prospectively followed 30 children with biopsy-confirmed EoE for a median of 14.5 months, documenting IgE sensitization patterns across all six canonical EoE dietary triggers and assessing QoL using validated instruments. Their key observation—that children on a one-to-two-food elimination diet reported better QoL than those with either no dietary restriction or more than two food eliminations—is nuanced and clinically actionable, pointing to a therapeutic “sweet spot” where dietary management meaningfully controls symptoms without imposing the quality-of-life burden of highly restrictive regimens. This paper addresses a gap between guideline-level evidence (which favors empirical elimination diets) and the lived experiences of families navigating both EoE and IgE-mediated allergy simultaneously.

Verelst et al. highlight an even more undercharacterized clinical entity: primary allergy to non-cow mammalian milk without concurrent cow’s milk allergy (CMA). Through a case series of three adult patients and a comprehensive literature review of 82 published cases, they establish that these allergies are rare, typically late-onset (mean age 8.6 years across the literature; adult onset in their series), and associated with high rates of severe reactions. The development of a streptavidin-based in-house ImmunoCAP™ assay for buffalo milk proteins illustrates the diagnostic creativity required when commercial tools are unavailable—a situation common with rare food allergens. In their three patients, the confirmation of isolated sensitization without cross-reactivity to cow’s milk suggests that species-specific epitopes drive primary sensitization in these cases, with implications for dietary counseling: such patients may safely consume cow’s milk and its derivatives.

Rounding out the collection, Singh et al. provide a narrative review of the gut microbiome in IgE-mediated food allergy. This contribution situates the preceding clinical papers within a broader biological framework: the gut microbiome shapes immune tolerance through short-chain fatty acid production, epithelial barrier maintenance, regulatory T cell differentiation, bile acid signaling, and modulation of the post-weaning immune reaction. The review catalogs emerging interventions—probiotics (particularly Lactobacillus rhamnosus GG for cow’s milk allergy), fecal microbiota transplantation (FMT), immune-supportive dietary strategies, and the interplay between oral immunotherapy (OIT) and the microbiome—while carefully delineating what remains unproven. Notably, baseline microbiome composition, rather than OIT-induced microbiome changes, appears to be the stronger predictor of sustained unresponsiveness.

## 2. Directions for Future Research

The papers in this Special Issue collectively point toward several priority areas for the next generation of research. First, standardization of OFC protocols and predictive biomarkers across centers and populations is urgently needed. The sIgE cut-offs reported by Klim et al. are population-specific and cannot be assumed to generalize; multicenter prospective studies that pool data from diverse European and non-European populations are required to derive more robust thresholds. Component-resolved diagnostics (CRD)—assessing individual allergen molecules such as casein, ovomucoid, and Ara h 2—merit systematic incorporation into OFC eligibility frameworks, particularly as evidence accumulates that molecular sensitization profiles more precisely stratify reaction risk than extract-based sIgE alone.

Second, Kucharek et al.’s null finding regarding BMI and anaphylaxis severity in children should stimulate prospective, adequately powered cohort studies with longer follow-up. Whether obesity begins to exert an amplifying effect on anaphylaxis severity during adolescence—as the physiological and metabolic transition toward an adult phenotype occurs—is a question with direct clinical relevance for anaphylaxis risk counseling in teenagers. Mechanistic studies examining adipokine levels, cardiac mast cell density, and inflammatory mediator profiles in obese versus non-obese children experiencing anaphylaxis would help to clarify whether the biological substrate for an obesity–severity link is present but has simply not yet clinically manifested in this age group.

Third, the EoE–IgE allergy overlap described by Nuyttens et al. demands larger, randomized trials to determine optimal treatment sequencing. The authors’ ongoing PedEoE IgE study (NCT06381219), exploring whether extensively heated cow’s milk or hen’s egg can be introduced safely in children with food-induced EoE, exemplifies the kind of carefully staged translational inquiry that this field needs. If tolerance to heated allergens can indeed be leveraged to progressively broaden the diet in these children without triggering EoE reactivation, this would represent a meaningful quality of life advancement. Future studies should also examine whether IgE sensitization complexity at diagnosis predicts long-term EoE outcomes, including time to remission and risk of esophageal fibrosis.

Fourth, the diagnosis and management of rare mammalian milk allergies requires concerted investment in standardized commercial diagnostics. The in-house assay developed by Verelst et al. for buffalo milk is an important proof of concept, but its widespread adoption in clinical laboratories is not feasible without validated commercial platforms. Collaborative European allergy registries—such as the EAACI Anaphylaxis Registry, which contributed data to this work—are well positioned to accumulate sufficient case numbers for systematic allergen characterization studies of buffalo, mare, donkey, and camel milk proteins.

Fifth, and perhaps most expansively, the microbiome review by Singh et al. charts a research agenda that has the potential to transform food allergy management over the next decade. Several priorities stand out: FMT trials for food allergy are still in their infancy; the results of the Phase I (NCT02960074) and Phase II (NCT05695261) trials for peanut allergy will be pivotal in determining whether this approach warrants larger investment. The immune-supportive diet proposed by Vlieg-Boerstra and colleagues requires rigorous RCT evaluation—an ongoing trial (NCT05667610) in children with peanut and/or nut allergy will provide preliminary data, but more extensive studies across diverse food allergies and geographic contexts will be necessary. Standardized microbiome endpoints for clinical trials—analogous to the role sIgE plays in conventional allergy trials—need to be developed and validated. Alpha-diversity, SCFA-producing taxa abundance, and bile acid profiles are plausible candidates, but consensus is lacking.

Finally, a theme running through all five papers is the value of longitudinal, real-world data over short-term controlled experiments. The natural history of food allergy—tolerance acquisition, persistence, and factors that modulate both—can only be fully characterized through follow-up spanning years to decades. Registries, biobanks, and electronic health record linkages offer practical vehicles for this kind of inquiry, and their integration with omics platforms (microbiome, metabolome, transcriptome) will be essential for moving from association to mechanism.

## 3. Conclusions

This Special Issue demonstrates that nutrition and diet are not peripheral to allergy care—they are central to it, from the specific protein fractions that trigger IgE-mediated reactions, to the dietary patterns that shape the gut microbiome and immune tolerance, to the quality-of-life burden imposed by therapeutic food restriction [1,3]. The five contributions published here advance clinical knowledge in areas that are immediately relevant to practitioners managing food-allergic patients: who can safely undergo an oral food challenge and when; whether body weight modifies risk in pediatric anaphylaxis; how to navigate the complex overlap between EoE and IgE food allergy; how to diagnose and counsel patients with rare mammalian milk allergies; and how the microbiome might eventually be harnessed as a therapeutic target.
